# Mechanical Transformation of Compounds Leading to Physical, Chemical, and Biological Changes in Pharmaceutical Substances

**DOI:** 10.1155/2018/8905471

**Published:** 2018-12-13

**Authors:** A. V. Syroeshkin, E. V. Uspenskaya, T. V. Pleteneva, M. A. Morozova, I. A. Zlatskiy, A. M. Koldina, M. V. Nikiforova

**Affiliations:** ^1^Peoples Friendship University of Russia (RUDN University), 6 Miklukho-Maklaya St., Moscow 117198, Russia; ^2^Dumanskii Institute of Colloid and Water Chemistry National Academy of Sciences of Ukraine, Kiev, Ukraine

## Abstract

This study demonstrates the link between the modification of the solid-phase pharmaceutical substances mechanical structure and their activity in waters with different molar ratio *«*deuterium-protium*»*. Mechanochemical transformation of the powders of lactose monohydrate and sodium chloride as models of nutrients and components of dosage forms was investigated by the complex of physicochemical and biological methods. The solubility and kinetic activity of substances dispersed in various ways showed a positive correlation with the solvent isotope profile. Substances dissolved in heavy water were more active than solutes in natural water. Differential IR spectroscopy confirmed the modification of substituents in the sample of lactose monohydrate, demonstrating physicochemical changes during mechanical intervention. The biological activity of the compounds was determined by the method of Spirotox. The activation energy was determined by Arrhenius. Compared with the native compound, dispersed lactose monohydrate showed lower activation energy and, therefore, greater efficiency. In conclusion, proposed data confirm the statement that mechanical changes in compounds can lead to physicochemical changes that affect chemical and biological profiles.

## 1. Introduction

To predict expected bioavailability characteristics for drug substances the pharmaceutical scientists give their significant attention to investigate the dependence of the pharmaceutical ingredient's activity on the degree of dispersion [1, 2].

There is direct relationship between the pharmaceutical substance dissolution rate in biological fluids and its bioavailability [3–6]. The increase in solubility and dissolution rate of substances that are poorly soluble in water promotes both their release from the dosage form and their penetration through the biological membranes [7, 8].

Mechanical treatment is one of the methods to activate physicochemical processes when studying the properties of active pharmaceutical ingredients (APIs) powders* in vitro *[9–11]. In the process of solids dispersing, the centers of increased activity appear on newly formed surfaces as a result of the accumulation of point defects, amorphous regions, structural changes, an increase in the specific surface area, and decrease of powder's average particle size [12–15].

The present work illustrates that the mechanical dispersing (grinding) and fluidization of solid pharmaceutical substances is accompanied by the change in their physicochemical and biological properties. The substances of different chemical and pharmacological classes were chosen as the research objects: lactose monohydrate (as the most common excipients used in pharmaceutical technology-diluents of tablets, capsules, and powders) [16] and sodium chloride (used in treatment of electrolyte deficiency and as osmotic agent in dosage forms) [17].

A unique way to change solubility of already known and new pharmaceutical substances is using the specified isotope composition of the dispersion medium [18–20]. We used as a solvent water with natural or modified D/H isotopic ratio. Due to the kinetic isotopic effect we were able to analyze the kinetic changes in solutions of powders dispersed in various ways in the samples of different waters [21].

Thus, the aim of the work was to study the influence of the mechanical preparation methods (grinding, fluidization) of solid pharmaceutical substances on their physicochemical properties and biological activities by assessing their dispersity (optic microscopy and laser diffraction), Fourier-transform infrared spectra (in the middle and terahertz regions), dissolution kinetics in waters of different D/H isotopic ratio, living cells survival kinetics, and Arrhenius kinetics.

## 2. Materials and Methods

### 2.1. Test Substances and Solvents

Lactose monohydrate C_12_H_22_О_11_·H_2_О (DFE Pharma, Germany). Sodium chloride NaCl (Sigma-Aldrich, USA). Solutions of the test substances were prepared by weighing on analytical scales ATL-80d4 (Acculab) and dissolution in water with a specific D/H ratio. The water with a modified hydrogen isotopic composition was used as a solvent -deuterium-depleted water (ddw) with D/H = 4 ± 0.9 ppm (Sigma-Aldrich, USA); deuterated water 99,9% D_2_O (Sigma-Aldrich, USA); deionized water (MilliQ) with the natural D/H ratio = 140 ± 0.9 ppm. The content of deuterium and oxygen-18 was controlled by multiple-pass laser absorption spectroscopy with an Isotopic Water Analyzer-912-0032 (Los Gatos Research, Inc., USA).

### 2.2. Powder Dispersion

Microstructuring of lactose monohydrate and sodium chloride was performed by grinding in a mechanical cutting knife mill in the form of “free direct strike” [22] for 10 minutes in isocratic mode. Lactose was also treated by the technique of applying an aqueous-alcoholic solution to the substance in the so-called fluidized bed (fluidization chamber).

The dispersity of pharmaceutical substances was analyzed by several methods: optical microscopy, laser diffraction, Spirotox method, and Fourier-transform infrared spectroscopy.

### 2.3. Optical Microscopy

Optical microscopy was used to determine the size and shape of crystalline substances particles, which are individual characteristics of the substance. The studies were carried out with the Altami BIO 2 microscope (Altami, Russia) with the 10xobjective magnification. A sample of substance was applied on a slide and spread over it so that the powder particles were in the same plane. The particles were observed in separate fields of view. For each series 10 fields were examined, and each field contained 6 to 30 particles. Then the size of the particles was measured using the “Altami Studio 3.3” program and a USB camera (3 Mpix resolution). The calibration was made using a micrometer object.

### 2.4. Laser Diffraction Method

Granulometric analysis (numerical and volume distribution of particles by size/volume spectra). Dissolution kinetics of powders with different dispersity were performed by the low-angle laser light scattering (LALLS) method at the Particle Sizer [23], using MasterSizer 3600 (MALVERN Instruments, UK) and Cluster-1-IDL-1, and laser dispersion meter (ICCWC-RUDN, Ukraine-Russia).

### 2.5. Fourier-Transform Infrared Spectroscopy

The analysis in the middle IR region was carried out using an IR Cary 630 Fourier spectrometer (Agilent Technologies, USA) with an ATR attachment with a diamond crystal. The instrument control, data measurement, and processing were performed with Agilent MicroLab Expert software. The results of terahertz spectrometry were obtained on an IR Fourier spectrometer Vertex 70 (Bruker, Germany), which was equipped with a vacuum pump and a mercury lamp.

### 2.6. Biological Activity of Pharmaceutical Substances (Biosensor Spirostomum ambigua)

The Spirotox method [24, 25] was used to determine the biological activity of the* S. ambigua *infusorium in the solutions of variously dispersed substances. The life-span of the biosensor was determined at different temperatures. The activation energy was calculated from the dependence of infusoria death rate constant on the reciprocal temperature (Arrhenius coordinates) [18]. For the stable medium temperature control and maintaining the experiment was carried out using Lauda A6 (Lauda, Germany) thermostat. The MBR-10 (Altami, Russia) binocular microscope was used for the biosensor monitoring. The biosensor was placed into the 5-well plate with the investigated substance solution and the death time was fixed counting from the moment of placing into the well.

### 2.7. Statistics

Origin 8 and MS Excel programs were used for the processing and statistical analysis of the experimental data. All results were expressed as a mean ± standard deviation (SD). Statistical analysis was performed by Student's t-test. All analyses were conducted at the 95% confident level; p < 0.05 was assumed as the statistically significant difference between the experimental points.

## 3. Results and Discussion

The industrial control over the technological characteristics of the original substances is necessary for the preparation of high-quality dosage forms. The influence of the shape and size of pharmaceutical substance particles on the technological characteristics of the tablet mass is of particular notice. If the drug's particles size is reduced to a nanometer range, the overall effective surface area increases and, thereby, the dissolution rate increases [2, 26].

### 3.1. Determination of the Size and Shape of Crystalline Substances Particles

Dispersity and the shape of the crystals of APIs and excipients are of high importance in the development of the solid dosage forms [1, 27]. Therefore it is advisable to estimate the size (distribution by fractions) and the shape of particles of the potential pharmaceutical substance's candidate already at the stage of the first screening.

**Figure 1:**
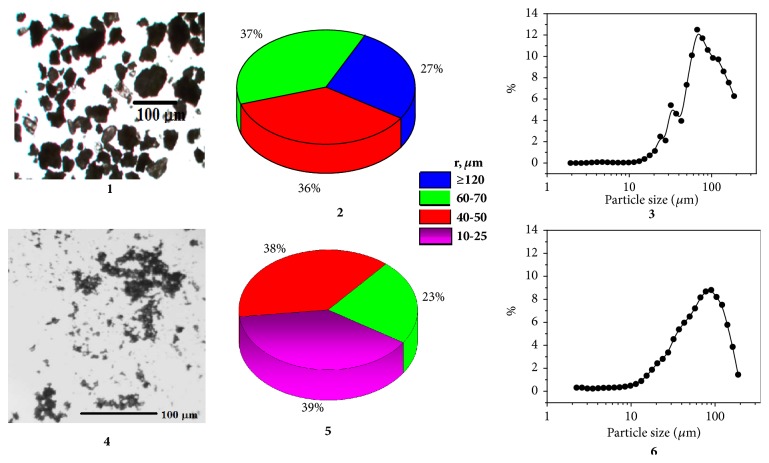
Particle size distribution (optical microscopy 1,2; 4,5 and laser diffraction 3,6) of native (1-3) and dispersed (4-6) lactose monohydrate powders (SuperTab 30G).* Granulometric analysis of the lactose monohydrate suspension by laser diffraction method (volume distribution, measurement interval from 1 to 120 μm) was carried out immediately after applying 0.6 g of the substance into 3 ml of water *(n ≥ 3, р < 0.05).

According to the microscopic examination, the particles of the native lactose monohydrate sample are anisodiametric (irregular) in shape, with a low bulk density. In the planar projection, the shape of the crystals can be brought to the following geometric forms: lamellar (the length and width are much greater than the thickness) and equiaxial particles (spherical, polyhedra, and the shape of which is close to the isodiametric), belonging to group II, according to the classification of substances based on the form of particles of the dominant fraction (Figure 1). Powders of group II have low flow ability and compressibility [28].

After treating the test substances, the particles with broken edges, coarse, and uneven surfaces appear, as well as signs of agglomeration. Microscopic analysis of a native lactose monohydrate sample showed that in particle size distributions about 27% of the particles have a size > 80 *μ*m. A large proportion (37%) falls on the size group 61-80 *μ*m; particles of 41-60 *μ*m in size are 27%; the remaining groups (<40 *μ*m) are less than 9%. After the 10-minute grinding mode, the lactose monohydrate particle's size decreased almost twice: only about 23% reached a maximum size of ~60 *μ*m. Specific surface area (m^2^/cm^3^) of the initial sample of lactose monohydrate was 0,0930 ± 0,0134 and after mechanical activation was 0,2204 ± 0,1429.

The histogram of the volume distribution of the particles in their native size has two maxima and two shoulders that characterize the polydispersity of the suspension. After dispersing, the volume fraction of particles of radius 90 *μ*m decreases from 13% to 9%. Asymmetric histogram has a smooth rise of the left branch and sharp drop of the right branch to almost zero. This type of volume distribution curve reflects the increase in number of dispersed particles and also the increase of monodispersity of solid phase. Large volume is occupied by some massive particles, what indicated possible agglomeration of small particles. The last was confirmed by the microscopic method.

Thus, it was shown that intensive mechanical treatment of substances led to an increase in the dispersity.

It was important to compare the rates of dissolution of the initial and dispersed samples in aqueous solutions with different D/H ratios.

### 3.2. Kinetics of Lactose Monohydrate Samples Dissolution after Mechanoactivation in Dependence on the Hydrogen Isotopic Composition of the Solvent

The consequences of kinetic isotope effects for the slowing down of biochemical reactions involving the heavier hydrogen isotope ^2^H (deuterium) are interesting for pharmacy [29, 30]. The pharmaceutical industry takes it into account to slow down drug metabolism processes [31]. This is because C-D bond is cleaved 6–10 times more slowly than a С-H bond. The water D/H isotopic ratio can significantly affect the kinetics and rate of chemical reactions [31, 32].

**Figure 2:**
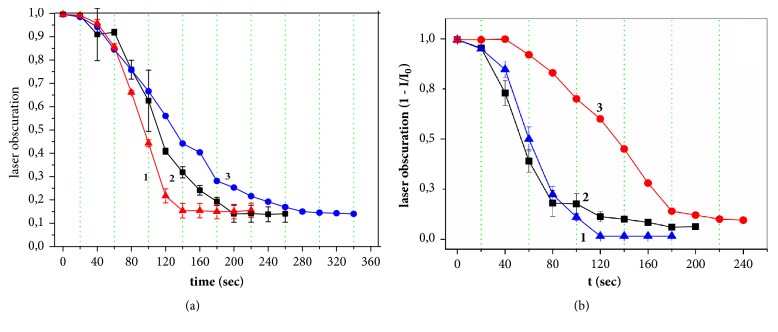
The laser obscuration values as solubility time function of native (a) and dispersed (b) lactose monohydrate in waters with the different D/H ratio: 1: ddw, 2: MiliQ, and 3: D_2_O.

Native and dispersed lactose monohydrate samples were used as models to study the effect of the deuterium/protium ratio in water on the solubility kinetics of APIs and excipients (Figure 2 and Table 1).

Decreased particles size as a result of mechanoactivation led to an increasing of solubility rate constants in comparison with native samples. The ratios of the solubility rate constants in natural water or ddw to heavy water was greater after substance dispersing.

Significant differences in the values of solubility rate constants of lactose monohydrate samples were established for solutions with different hydrogen heavy isotope contents. It was shown that solubility constants of native and dispersed lactose monohydrate in ddw are about a half times higher than in natural water and 3-4 times higher than the solubility rate in heavy water. This indicates the existence of an isotope kinetic effect for the dissolution process.

### 3.3. Difference between Lactose Samples Assessed by IR Fourier Spectroscopy

A study using IR Fourier spectroscopy in the middle and terahertz regions revealed significant differences between initial sample and the sample after fluidization.

**Figure 3:**
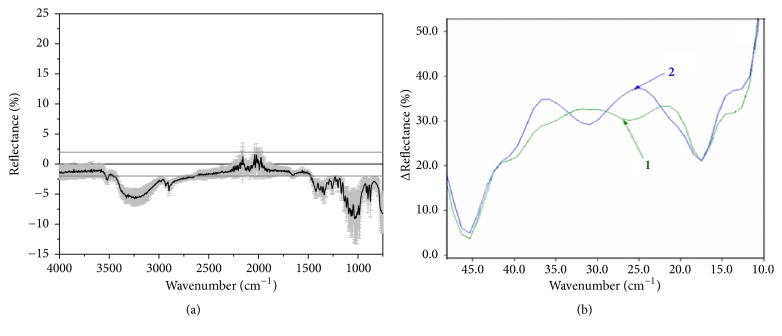
Differential IR spectra with the means of standard deviation (gray area) in the middle range (a) and in the terahertz range (b) for the sample of the native lactose (1) and lactose after the fluidization chamber (2).* ΔReflectance = Reflectance of native sample – Reflectance of the sample after fluidization chamber*.

It should be emphasized that the sample after the fluidization chamber differs sharply from the initial one (Figure 3), for instance, by the absence of a maximum at 25 cm^−1^. It should be also noted that in the shorter-wave region up to 4000 cm^−1^, in the middle IR range, we also found significant differences between the samples. So, it can be concluded that the differences between lactose samples identified in the far and middle IR range are due to differences in the organization of supramolecular lactose structures in the amorphous and quasi-crystalline state.

### 3.4. Determination of the Biological Activity of Powders with Different Dispersity by the Spirotox Method

**Figure 4:**
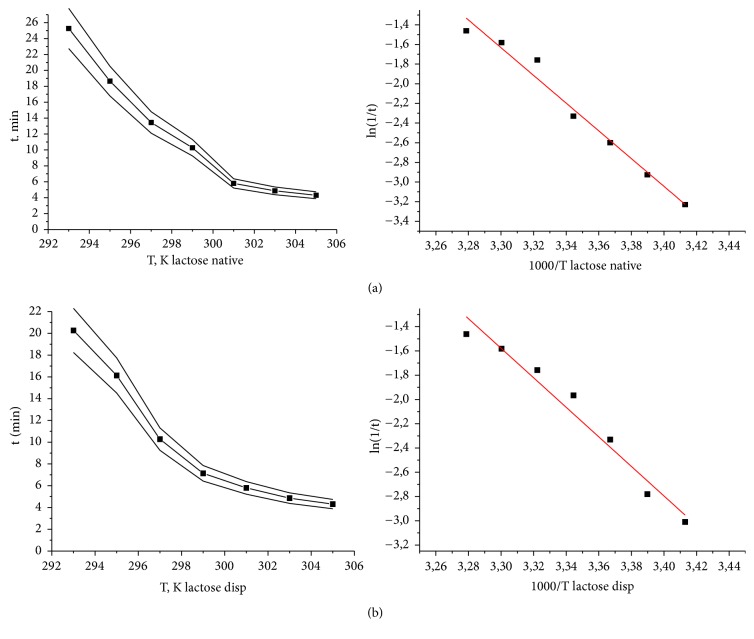
Dependence of the infusoria mortality rate on temperature and dispersity for the aqueous solutions of 7% lactose monohydrate in direct (left graph) and Arrhenius coordinates (right graph). (a) Native lactose and (b) dispersed lactose. n ≥ 5; р < 0,05.

**Figure 5:**
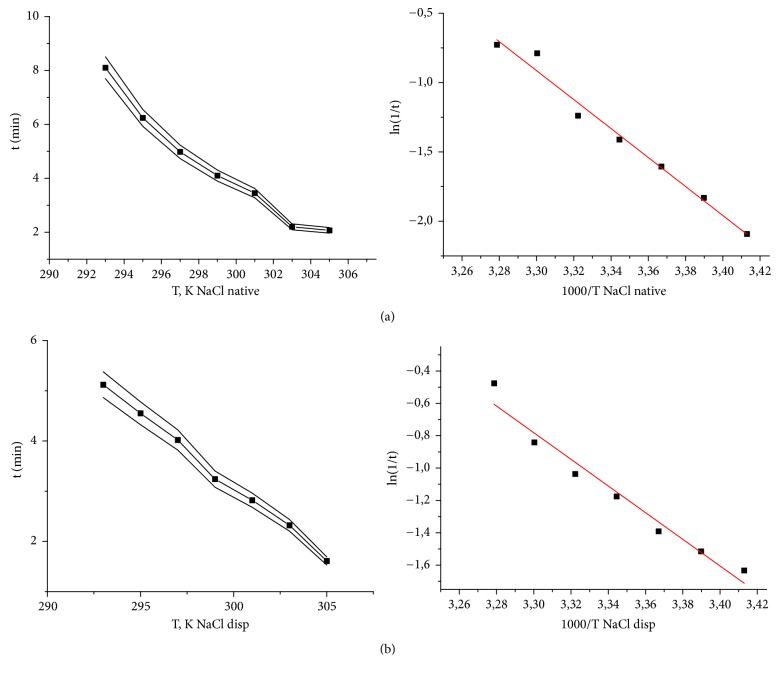
Dependence of the infusoria mortality rate on temperature and dispersity for the aqueous solutions of 1% sodium chloride in direct (left graph) and Arrhenius coordinates (right graph). (a) Native sodium chloride and (b) dispersed sodium chloride. n ≥ 5; р < 0.05.

Sodium chloride was taken as a comparison sample to exclude the influence of the crystalline substance nature on the effect of the lactose monohydrate mechanical activation. A study of the temperature dependence for the ligand-induced death of test objects in 7% aqueous solutions of lactose monohydrate and 1% sodium chloride was carried out in a temperature range of 24-36°C with 2°C intervals. For the solutions of all investigated compounds the exponential dependence of the infusoria mortality rate was characteristic, irrespective of the produced effect (Figures 4 and 5) [33, 34].

It was found that the dependence between lifetime and the temperature of ligand-induced cell transitions of* S. ambiguum *is linearized in Arrhenius coordinates: lg⁡(1/t)-1000/T. The values of the apparent activation energy (obsEa) were found by the tangent of the lines slope in semilogarithmic coordinates (Table 2).

The effect of mechanochemical activation of pharmaceutical substances on biological activity was confirmed by the Arrhenius kinetic regularities. Statistically significant differences in the activation energies of solutions of native and dispersed samples were obtained. The analysis of the Arrhenius kinetics results testifies the so-called “structural factor” of the reaction acceleration [35]. The time of ligands (pharmaceutical substances) mass transfer to the cellular receptor decreases, the reaction activity, and the reaction rate increase (Figures 4 and 5; Table 2). The observed increase in the influence of the dispersed substance on the biosensor is quantitatively estimated by the values of the activation energy obsEa. It should be noted that the decrease in the activation energy after dispersion is characteristic for both the substances of organic (lactose) and inorganic (sodium chloride) origin.

## 4. Conclusions

The present work describes the presence of dimensional and surface effects of pharmaceutical substances in the system “dispersion-structure-properties”. This study demonstrates the relationship between the modification of the mechanical treatment of solid-phase substance and its solubility rates (laser diffraction method) in the waters of different deuterium/protium ratio (kinetic isotope effect), IR spectral properties in the middle and terahertz ranges, and differences in ligand-receptor interactions as a function of the dispersity of pharmaceutical substances samples for the biological model (Spirotox, the Arrhenius interpretation).

## Figures and Tables

**Table 1:** The lactose monohydrate solubility rate constants and kinetic isotopic effect in water with different D/Н ratios for native and dispersed substances.

Lactose monohydrate sample	$(\bar{k}\pm \Delta \bar{k)}$ ***∙*10** ^**2.**^ **, s** ^**-1**^ n = 3, Р = 95%		**k** ***∙*10** ^**2**^ **, s** ^**-1**^ n = 1	$\frac{k_{ddw}}{k_{MilliQ}}$	$\frac{k_{ddw}}{k_{D2O}}$	$\frac{k_{MilliQ}}{k_{D2O}}$
	ddw D/H = 4 ± 0.9 ppm	MilliQ D/H = 140 ± 0.9ppm	D_2_O 99,9 %			
Native substance	2.25 ± 0.089	1.36 ± 0.144	0.80	1.6	2.8	1.7

Dispersed substance	3.46 ± 0.199	2.26 ± 0.215	0.88	1.5	3.9	2,6

**Table 2:** The *S. ambiguum* ligand-induced death activation energy for the lactose monohydrate and sodium chloride samples solutions (n = 15, ^*∗*^p < 0,05).

Sample	Activation energy obsEa, kJ/mol	
	Native substance (Mean ± SD)	Dispersed substance (Mean ± SD)
Lactose monohydrate	117 ± 0.93	101 ± 1.0
Sodium chloride	87 ± 1.0	68 ± 1.0

## Data Availability

The data used to support the findings of this study are available from the corresponding author upon request.
